# Supplementary material for: Silver lining to a climate crisis in multiple prospects for alleviating crop waterlogging under future climates
Source: Nat Commun. 2023 Feb 10;14:765. doi: 10.1038/s41467-023-36129-4 (PMC9918449; doi:10.1038/s41467-023-36129-4)
Supplement: Supplementary file 1 — Supplementary Information [file 41467_2023_36129_MOESM1_ESM.pdf]

Supplementary information

Silver lining to a climate crisis: multiple prospects for alleviating crop waterlogging under future climates

Supplementary Tables 1-4

Supplementary Figures 1-12

Supplementary Table 1 Parameterised APSIM genotypic coefficients of genotypes used in waterlogging experiments (Exps). Abbreviations: tt_end_of_juvenile (TEJ, thermal time from sowing to end of juvenile stage), tt_start_grain_fill (TSGF, thermal time at the beginning of grain filling), photop_sens (PPD, photoperiod sensitivity), vern_sens (VERN, vernalisation sensitivity), grains_per_gram_stem (GPGS, the number of grain per gram of stem), potential_grain_filling_rate (PGFR, grain growth rate during grain-filling stage). Measured data from experiments conducted in five countries were used for model development and evaluation. Exp1 was conducted under controlled conditions (Launceston, Tasmania, Australia) with four waterlogging treatments using six contemporary Australian barley genotypes differing in their waterlogging tolerance from 2019 to 2020 (see ref.[1, 2]). In Exp2, barley yields were measured under five waterlogging treatments in the greenhouse and field conditions at the School of Agronomy, University of Buenos Aires, Argentina during 2010 (see ref.[3]). In Exp3, barley genotypes were evaluated for waterlogging tolerance in controlled field conditions at Brandon Research and Development Centre, Brandon, Manitoba, Canada from 2016 to 2017. Waterlogging treatments were initiated at the tillering stage by adding the water to heights of 0.5–1 cm above the soil surface (see ref.[4]). In Exp4, barley yields were measured in field conditions carried out at Oak Park, Carlow, Ireland from 2017 to 2018. Waterlogging treatments were initiated at the tillering stage using a boom irrigator (see ref.[5]). In Exp5, field experiments were conducted in 2003-2004 and 2005-2006 at Zhejiang University, Hangzhou, China (see ref.[6, 7]).

**Exps**	**Genotypes**	**TEJ (°C)**	**TSGF (°C)**	**VERN**	**PPD**	**GPGS (g)**	**PGFR (g grain^{-1} d^{-1})**	**oxdef_photo rtfr**	**oxdef_photo**	**x_oxdef_stage _photo**	**y_oxdef_lim _photo**
Exp1	Macquarie	873	410	1	0.5	27.5	0.0029	0, 0.8, 1.0	1.0, 1.0, 0.8	4.179, 4.271, 4.382, 5.667	0.03, 0.95, 0.72, 0
Exp1	Macquarie (T）	870	428	1	0.5	27.5	0.0025	0, 0.8, 1.0	1.0, 1.0, 0.8	4.179, 4.271, 4.382, 5.667	0.38, 0.90, 0.87, 0
Exp1	Planet	590	440	1	2.3	28.8	0.003	0, 0.8, 1.0	1.0, 1.0, 0.8	4.179, 4.271, 4.382, 5.667	0.03, 0.99, 0.80, 0
Exp1	TamF169	620	430	1	2.1	28.0	0.0027	0, 0.8, 1.0	1.0, 1.0, 0.8	4.179, 4.271, 4.382, 5.667	0.3, 0.93, 0.84, 0
Exp1&Exp5	Franklin	710	410	1.5	2.2	27.0	0.0026	0, 0.8, 1.0	1.0, 1.0, 0.8	4.179, 4.271, 4.382, 5.667	0.04, 0.95, 0.75, 0
Exp1	Westminster	890	410	1	0.2	27.7	0.003	0, 0.8, 1.0	1.0, 1.0, 0.8	4.179, 4.271, 4.382, 5.667	0.03, 0.95, 0.73, 0
Exp2	Scarlett	590	540	0.2	2.4	28.8	0.003	0, 0.8, 1.0	1.0, 1.0, 0.8	4.5, 4.8, 5.0, 6.0, 7.0	0.7, 0.65, 0.60
Exp3	PI370983	265	340	0.3	2.9	28.2	0.0028	0, 0.8, 1.0	1.0, 1.0, 0.8	5.0, 5.467	0.031, 0.011
Exp3	PI371100	250	320	0.1	3.1	25	0.0027	0, 0.8, 1.0	1.0, 1.0, 0.8	5.0, 5.467	0.033, 0.013
Exp3	PI573617	270	380	0.2	3.6	33	0.0027	0, 0.8, 1.0	1.0, 1.0, 0.8	5.0, 5.467	0.032, 0.014
Exp3	TX9425	230	180	1	3.1	35	0.003	0, 0.8, 1.0	1.0, 1.0, 0.8	5.0, 5.467	0.033, 0.012
Exp3	PI349896	300	320	0.5	1	29.1	0.003	0, 0.8, 1.0	1.0, 1.0, 0.8	5.0, 5.467	0.032, 0.011
Exp3	PI498439	295	330	0.1	0.8	29.8	0.0029	0, 0.8, 1.0	1.0, 1.0, 0.8	5.0, 5.467	0.034, 0.013
Exp4	Arma	690	550	4.2	3.3	30.1	0.0028	0, 0.8, 1.0	1.0, 1.0, 0.8	4.025, 4.076, 4.108	0.03, 0.61, 0.70
Exp4	Louise	610	590	4.5	3.2	32.5	0.0025	0, 0.8, 1.0	1.0, 1.0, 0.8	4.025, 4.076, 4.108	0.04, 0.65, 0.72
Exp4	Masquerade	650	570	3.5	3.2	33.2	0.0029	0, 0.8, 1.0	1.0, 1.0, 0.8	4.025, 4.076, 4.108	0.03, 0.60, 0.70
Exp4	Merode	650	565	4.8	3.8	29.8	0.003	0, 0.8, 1.0	1.0, 1.0, 0.8	4.025, 4.076, 4.108	0.05, 0.65, 0.74
Exp4	Portrait	625	555	4.2	3.5	33.1	0.0032	0, 0.8, 1.0	1.0, 1.0, 0.8	4.025, 4.076, 4.108	0.03, 0.62, 0.70

Supplementary Table 2. Global circulation models (GCMs) used to project Shared Socioeconomic Pathways Scenario SSP585 in this study.

Model ID	Name of GCM	GCM abbreviation	Institute ID	Country
1	ACCESS-CM2	ACC1	CSIRO-ACCESS	Australia
2	ACCESS-ESM1-5	ACC2	CSIRO-ACCESS	Australia
3	BCC-CSM2-MR	BCCC	BCC	China
4	CanESM5	Can1	CCCma	Canada
5	CanESM5-CanOE	Can2	CCCma	Canada
6	CIESM	CIES	THU	China
7	CMCC-CM2-SR5	CMCS	CMCC	Italy
8	CNRM-CM6-1	CNR2	CNRM-CERFACS	France
9	CNRM-CM6-1-HR	CNR3	CNRM-CERFACS	France
10	CNRM-ESM2-1	CNR1	CNRM-CERFACS	France
11	EC-Earth3	ECE1	EC-EARTH-Consortium	Europe
12	EC-Earth3-Veg	ECE2	EC-EARTH-Consortium	Europe
13	FGOALS-g3	FGOA	CAS	China
14	GFDL-CM4	GFD1	NOAA-GFDL	USA
15	GFDL-ESM4	GFD2	NOAA-GFDL	USA
16	GISS-E2-1-G	GISS	NASA-GISS	USA
17	HadGEM3-GC31-LL	HafG	MOHC	UK
18	INM-CM4-8	INM1	INM	Rusia
19	INM-CM5-0	INM2	INM	Rusia
20	IPSL-CM6A-LR	IPSL	IPSL	France
21	MIROC6	MIR1	MIROC	Japan
22	MIROC-ES2L	MIR2	MIROC	Japan
23	MPI-ESM1-2-HR	MPI1	MPI-M	Germany
24	MPI-ESM1-2-LR	MPI2	MPI-M	Germany
25	MRI-ESM2-0	MTIE	MRI	Japan
26	NESM3	NESM	NUIST	China
27	UKESM1-0-LL	UKES	MOHC	UK

Supplementary Table 3 APSIM phenology parameters for genotypes sown at each location under early (ESD) and late (LSD) sowing. Parameters were chosen to ensure that flowering occurred during the typical flowering period for the location assuming current practice. Parameters 'vern' and 'ppd' refer to vernalisation and photoperiod respectively, representing cumulative cold temperature requirement to initiate reproductive development and sensitivity to day length; higher values denote greater sensitivity. Parameter 'tt_emerg_to_endjuv' refers to the thermal time between emergence and the end of the juvenile phase (°C), another key determinant of development.

**Country**	**Sub-region**	**Abbrv.**	**Lat**	**Long**	**Soil type**	**Maturity type**	**tt_emerg_ to_endjuv (°C)**	**vern**	**ppd**	**ESD (DOY)**	**LSD (DOY)**
UK	Norwich	UKNo	52.68	1.31	Haplic Cambisols	Spring	400	1	1	61	122
UK	Burghed Beach	UkBu	57.69	-3.47	Haplic Cambisols	Spring	400	1	1	61	122
UK	Arborath	UKAr	56.59	-2.7	Endoleyic Cambisols	Spring	400	1	1	61	122
UK	Duns	UKDu	55.77	-2.34	Endoleyic Cambisols	Spring	400	1	1	61	122
Spain	Leon	ESle	42.67	-5.59	Haplic Luvisols	Spring	400	1	1	61	122
Spain	Cuenca	ESCu	40.21	-2.14	Halic Kasanozems	Spring	400	1	1	61	122
France	Arras	FRAr	50.37	2.63	Haplic Luvi sols	Spring	400	1	1	46	102
France	Merz	FRMe	49.24	6.14	Haplic Cambisols	Spring	400	1	1	46	102
Germany	Weiden	DEWe	49.61	12.11	Haplic Cambisols	Spring	400	1	1	62	131
Germany	Munich	DEMu	48.31	11.58	Haplic Cambisols	Spring	400	1	1	62	131
Ethiopia	Holetta	ETHo	9.05	38.48	Haplic Luvisols	Spring	400	1	1	153	243
USA	Lewistown	USLe	47.09	-109.46	Halic Kasanozems	Spring	400	1	1	61	151
USA	Logan	USLo	41.8	-111.92	Halic Kasanozems	Spring	400	1	1	61	151
USA	Rugby	USRu	48.42	-99.98	Halic Chernozems	Spring	400	1	1	61	151
Canada	Lethbridge	CALe	49.81	-112.74	Halic Kasanozems	Spring	400	1	1	122	182
Canada	Saskatoon	CASa	52.25	-106.65	Halic Chernozems	Spring	400	1	1	122	182
Russia	Suzemka	RUSu	52.31	34.07	Halic Albeluvisols	Spring	400	1	1	92	122
Russia	Morozovski	RUMo	48.38	41.7	Halic Chernozems	Spring	400	1	1	92	122
Russia	Tambov	RUTa	52.73	41.44	Halic Chernozems	Spring	400	1	1	92	122
Russia	Livny	RULi	52.39	37.58	Halic Chernozems	Spring	400	1	1	92	122
Australia	Wagga Wagga	AUWa	-34.83	147.49	Haplic Luvisols	Winter	400	4	1	92	172
Australia	Campbell Town	AUCa	-41.95	147.59	Haplic Cambisols	Winter	400	4	1	92	172
Argentina	Sierra de La Ventana	ARSi	-38.13	-61.79	Haplic Luvisols	Spring	400	1	4	162	223
Argentina	Loberia	ARLo	-38.1	-58.83	Luvic Phaeozems	Spring	400	1	4	162	223
China	Yancheng	CNYa	33.43	120.14	Haplic Fluvisols	Winter	400	4	2.5	294	330
China	Huaian	CNHu	33.62	119.12	Haplic Fluvisols	Winter	400	4	2.5	294	330
Germany	Weiden	DEWe	49.61	12.11	Haplic Cambisols	Winter	660	4	2.8	245	306
Germany	Munich	DEMu	48.31	11.58	Haplic Cambisols	Winter	660	4	2.8	245	306
Russia	Metelev	RUMe	46.88	39.07	Halic Chernozems	Winter	500	4	3	306	335

Russia	Stavropol	RUSt	45.12	41.82	Halic Chernozems	Winter	500	4	3	306	335
UK	Norwich	UKNo	52.68	1.31	Haplic Cambisols	Winter	690	4	3	259	306
UK	Burghed Beach	UkBu	57.69	-3.47	Haplic Cambisols	Winter	690	4	3	259	306
UK	Arborath	UKAr	56.59	-2.7	Endoleyic Cambisols	Winter	690	4	3	259	306
UK	Duns	UKDu	55.77	-2.34	Endoleyic Cambisols	Winter	690	4	3	259	306
Ukraine	Kharkiv	UAKh	50.00	36.26	Halic Chernozems	Spring	400	1	4	101	141
Ukraine	Mykolaiv	UAMy	47.07	32.06	Halic Chernozems	Spring	400	2.5	4	70	92
Australia	Esperance	AUEs	-33.59	121.87	Haplic Luvisols	Spring	400	2.5	4	92	172
Australia	Yarloop	AUYa	-32.95	115.9	Haplic Solonetz	Spring	400	2.5	4	92	172
Australia	Mortana	AUMo	-33.01	134.43	Haplic Solonetz	Spring	400	2.5	4	92	172
Australia	Minnipa	AUMi	-32.72	135.21	Haplic Arenosols	Spring	400	2.5	4	92	172
Australia	Cummins	AUCu	-34.15	135.79	Haplic Solonetz	Spring	400	2.5	4	92	172
Australia	Clare	AUCl	-33.75	138.68	Haplic Solonetz	Spring	400	2.5	4	92	172
Spain	Leon	ESle	42.67	-5.59	Haplic Luvisols	Winter	400	4	4	306	366
Spain	Cuenca	ESCu	40.21	-2.14	Halic Kasanozems	Winter	400	4	4	306	366
Turkey	Konya	TUKo	37.94	32.49	Haplic Calcisols	Winter	400	4	4	275	335
Turkey	Sanliurfa	TUSa	37.16	38.79	Calcic Vertisols	Winter	400	4	4	275	335
France	Arras	FRAr	50.37	2.63	Haplic Luvisols	Winter	750	4	4	245	306
France	Merz	FRMe	49.24	6.14	Haplic Cambisols	Winter	750	4	4	245	306

Supplementary Table 4. Change in yield of winter and spring barley for the 2040s (2030-2059) and 2080s (2070-2099) relative to the baseline (1985-2016) under early (ES) or late sowing (ES) for SSP585. Values are averaged for each climate period (2040s and 2080) across 27 GCMs.

Barley type	Country	Site	2040s		2080s	
			ES	LS	ES	LS
Spring barley	Argentina	Loberia	13%	10%	7%	14%
	Argentina	Sierra de La Ventana	13%	-4%	10%	-4%
	Australia	Clare	1%	0%	8%	5%
	Australia	Cummins	4%	2%	4%	3%
	Australia	Esperance	1%	4%	2%	8%
	Australia	Minnipa	2%	0%	2%	2%
	Australia	Mortana	0%	0%	0%	5%
	Australia	Yarloop	3%	6%	7%	21%
	Canada	Lethbridge	1%	-1%	19%	-1%
	Canada	Saskatoon	6%	-3%	16%	-3%
	Germany	Munich	5%	11%	13%	21%
	Germany	Weiden	6%	6%	16%	32%
	Spain	Cuenca	2%	14%	14%	45%
	Spain	Leon	3%	2%	5%	1%
	Ethiopia	Holetta	-1%	-12%	9%	-25%
	France	Arras	26%	22%	38%	38%
	France	Merz	12%	2%	23%	25%
	Russia	Livny	11%	6%	22%	22%
	Russia	Morozovski	7%	23%	26%	37%
	Russia	Suzemka	4%	3%	11%	14%
	Russia	Tambov	26%	19%	41%	56%
	Ukraine	Kharkiv	7%	8%	16%	36%
	Ukraine	Mykolaiv	-1%	26%	9%	64%
	United Kingdom	Arborath	3%	0%	1%	3%
	United Kingdom	Burghed Beach	2%	9%	-1%	13%
	United Kingdom	Duns	3%	14%	8%	22%
	United Kingdom	Norwich	2%	3%	8%	8%
	United states	Lewistown	4%	7%	5%	21%
	United states	Logan	-5%	21%	-7%	53%
	United states	Rugby	-0.2%	6.3%	-1.9%	15.7%
Winter barley	Australia	Campbell Town	16%	8%	24%	30%
	Australia	Wagga Wagga	4%	1%	8%	6%
	China	Huaian	4%	4%	3%	8%
	China	Yancheng	3%	-4%	12%	1%
	Germany	Munich	49%	17%	54%	20%
	Germany	Weiden	12%	9%	56%	5%
	Spain	Cuenca	14%	7%	26%	21%
	Spain	Leon	2%	-4%	14%	2%
	France	Arras	8%	4%	5%	10%
	France	Merz	50%	0%	75%	-5%
	Russia	Metelev	3%	7%	22%	7%
	Russia	Stavropol	35%	10%	82%	28%
	Turkey	Konya	22%	16%	26%	18%
	Turkey	Sanliurfa	-2%	1%	2%	9%
	Ukraine	Arborath	11%	3%	20%	7%
	Ukraine	Burghed Beach	9%	4%	23%	10%
	United Kingdom	Duns	19%	17%	20%	7%
	United Kingdom	Norwich	26%	19%	39%	11%

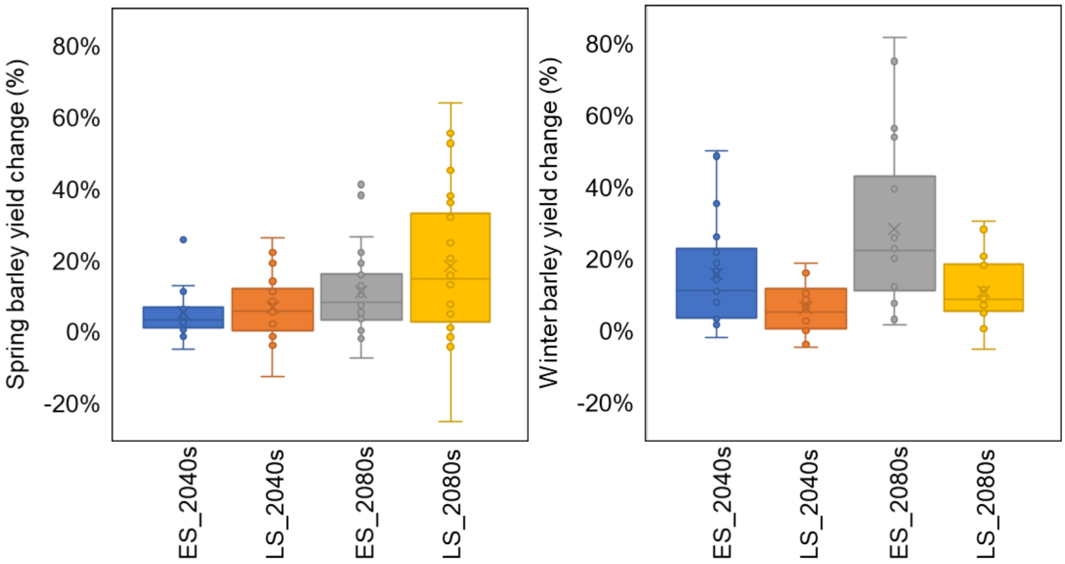

Supplementary Fig. 1 | Change in yield of winter and spring barley for 2040 (2030-2059) and 2080 (2070-2099) relative to the baseline period of 1985-2016 under early (ES) or late sowing (LS) for SSP585. Yield projections were simulated with a modified version of APSIM using climate projections from 27 GCMs. Boxplots indicate simulated yield change across sites and GCMs; box boundaries indicate 25th and 75th percentiles, and whiskers below and above the box indicate the 10th and 90th percentiles.

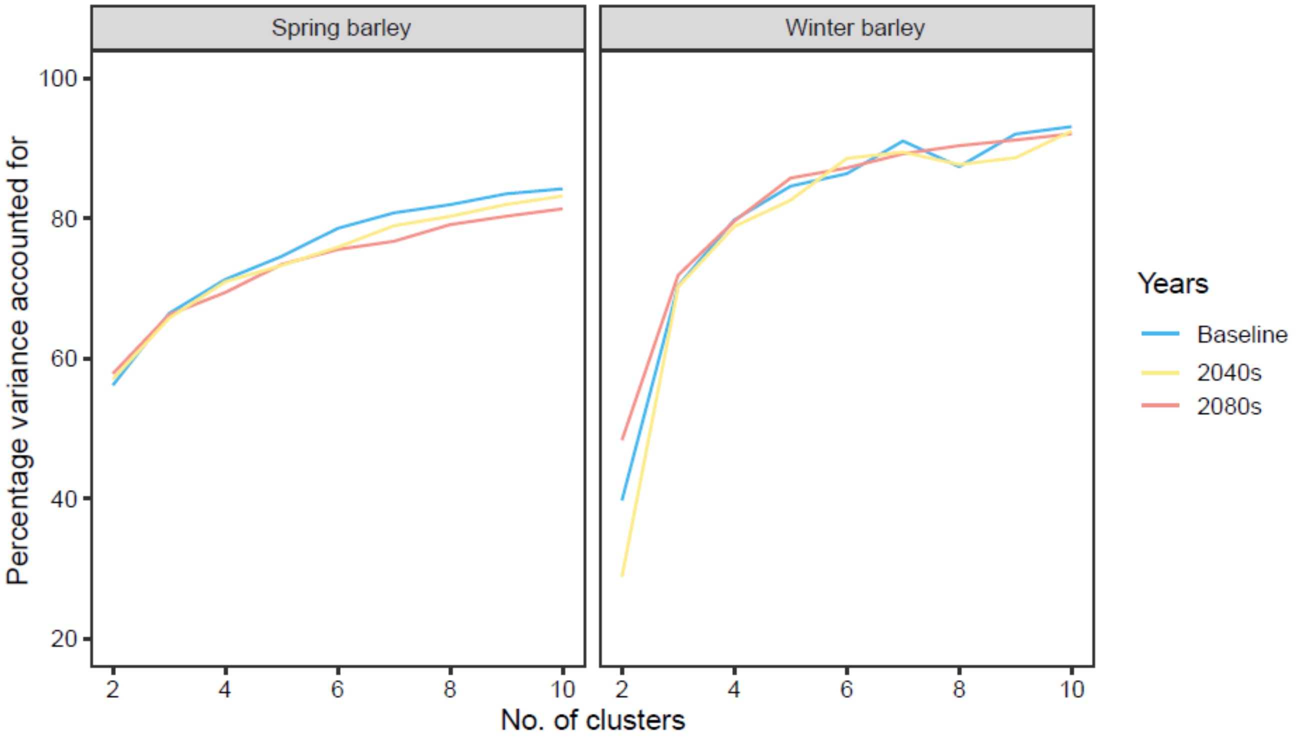

Supplementary Fig. 2| Percentage variance accounted for by *k*-means clusters for the baseline (1985-2016), 2040 (2030-2059) and 2080 (2070-2099).

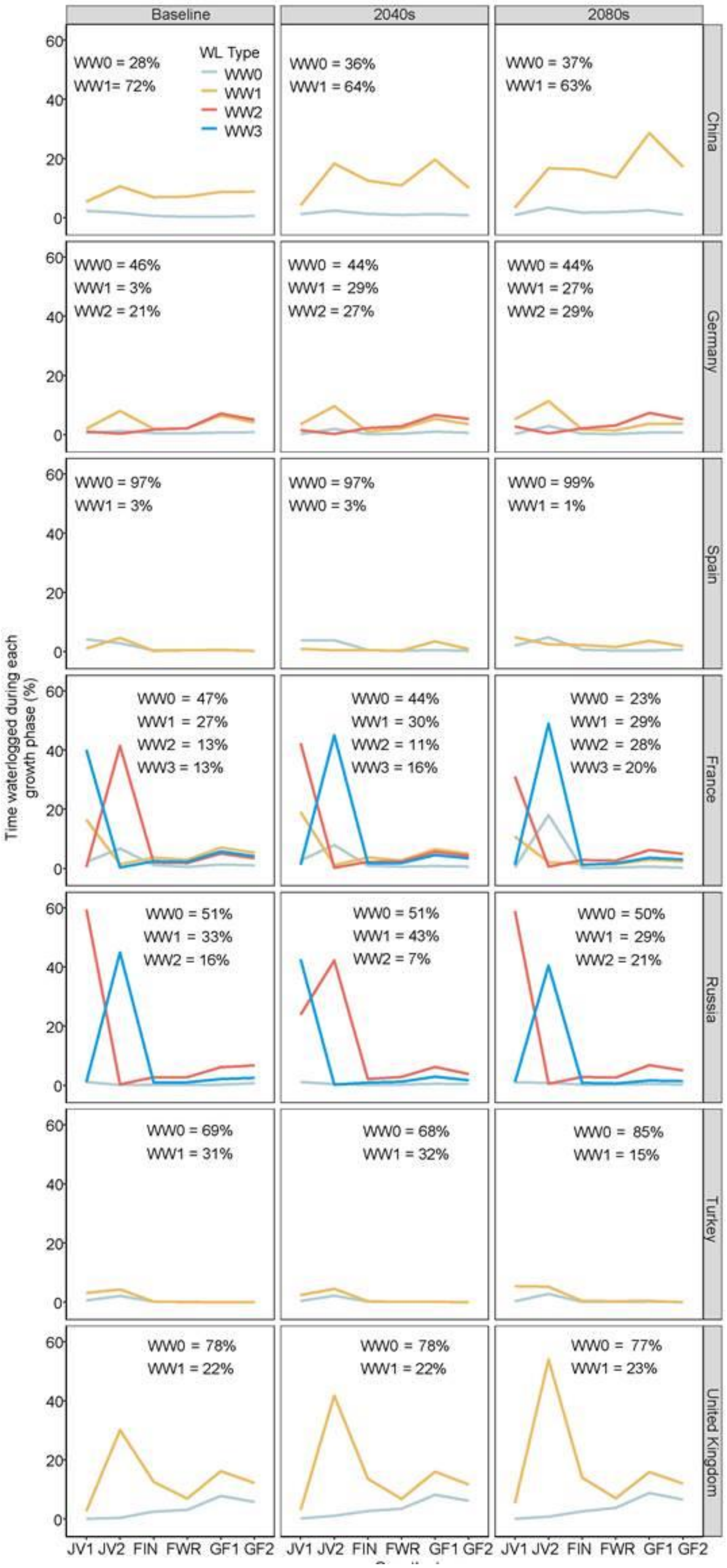

Baseline
2040s
2080s
China
Germany
Spain
France
Russia
Turkey
United Kingdom
WL Type
WW0
WW1
WW2
WW3
Time waterlogged during each growth phase (%)
WW0 = 28%
WW1= 72%
WW0 = 36%
WW1 = 64%
WW0 = 37%
WW1 = 63%
WW0 = 46%
WW1 = 3%
WW2 = 21%
WW0 = 44%
WW1 = 29%
WW2 = 27%
WW0 = 44%
WW1 = 27%
WW2 = 29%
WW0 = 97%
WW1 = 3%
WW0 = 97%
WW0 = 3%
WW0 = 99%
WW1 = 1%
WW0 = 47%
WW1 = 27%
WW2 = 13%
WW3 = 13%
WW0 = 44%
WW1 = 30%
WW2 = 11%
WW3 = 16%
WW0 = 23%
WW1 = 29%
WW2 = 28%
WW3 = 20%
WW0 = 51%
WW1 = 33%
WW2 = 16%
WW0 = 51%
WW1 = 43%
WW2 = 7%
WW0 = 50%
WW1 = 29%
WW2 = 21%
WW0 = 69%
WW1 = 31%
WW0 = 68%
WW1 = 32%
WW0 = 85%
WW1 = 15%
WW0 = 78%
WW1 = 22%
WW0 = 78%
WW1 = 22%
WW0 = 77%
WW1 = 23%
JV1 JV2 FIN FWR GF1 GF2

Supplementary Fig. 3| Waterlogging stress patterns and frequencies for the baseline period (1985-2016), 2040 (2030-2059) and 2080 (2070-2099) across countries and sowing dates. Waterlogging stress patterns for winter barley include (WW0: low waterlogging; WW1: low early-onset waterlogging relieved later; WW2: moderate early-onset waterlogging; WW3: severe early-onset waterlogging) and were identified by clustering seasonal time-courses of waterlogging stress across years, sowing dates, genotypes and countries. Growth stages include early juvenile development (JV1, 10<=APSIM growth stage<21); late juvenile development (JV2, 21<=APSIM growth stage<32); floral initiation to heading (FIN, 32<=APSIM growth stage<65); flowering to grain filling (FIN, 65<=APSIM growth stage<71; early grain filling (GF1, 71<=APSIM growth stage<80); late grain filling (GF2, 80<=APSIM growth stage<87).

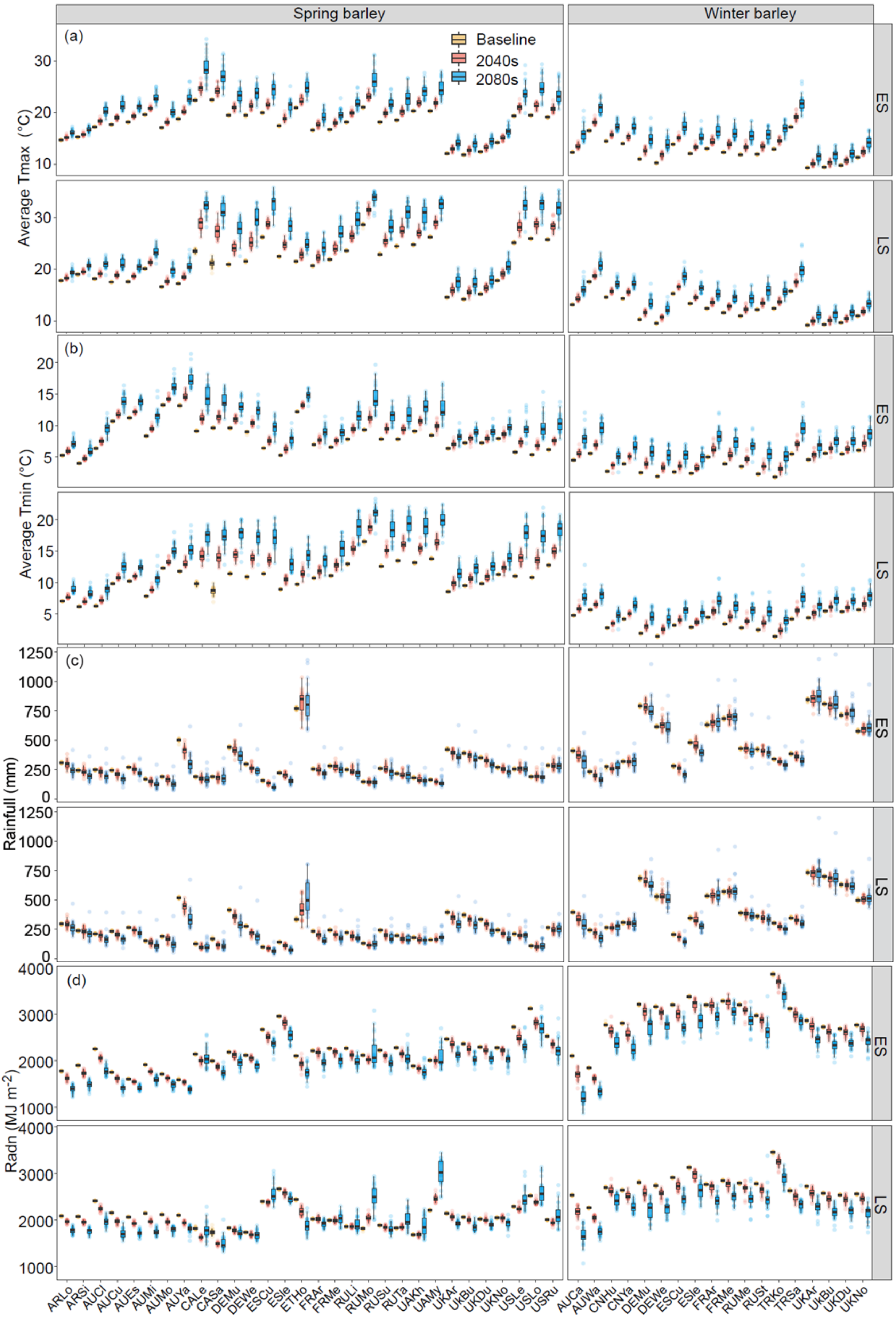
Spring barley
Winter barley
(a)
(b)
(c)
(d)
Baseline
2040s
2080s
ES
LS
Average Tmax (°C)
Average Tmin (°C)
Rainfull (mm)
Radn (MJ m-2)
ARLo ARSi AUCl AUCu AUEs AUMi AUMo AUYa CALe CASa DEMu DEWe ESCu ESle ETHo FRAr FRMe RULi RUMo RUSu RUTa UAKh UAMy UKAr UkBu UKDu UKNo USLe USLo USRu
AUCa AUWa CNHu CNYa DEMu DEWe ESCu ESle FRAr FRMe RUMe RUSt TRKo TRSa UKAr UkBu UKDu UKNo

Supplementary Fig. 4| Projected changes in temperature, rainfall and solar radiations projected from 27 GCMs for SSP585 in 2040 (2030-2059) and 2080 (2070-2099) compared with measured baseline values (1985-2016) at each site. a-d, maximum temperature (**a**)**,** minimum temperature (**b**), growing season rainfall (**c**), and growing season solar radiation (**d**). Boxplots show the 10th, 25th, 75th and 90th percentiles. Full names of the 27 GCMs are shown in Supplementary Table 4; ESD and LSD refer to early sowing and late sowing dates, respectively. Labels on the x-axis are abbreviations depicting country name concatenated with site name e.g. ARLo = Loberia, Argentina; ARSi = Sierra de La Ventana, Argentina; see Supplementary Table 1.

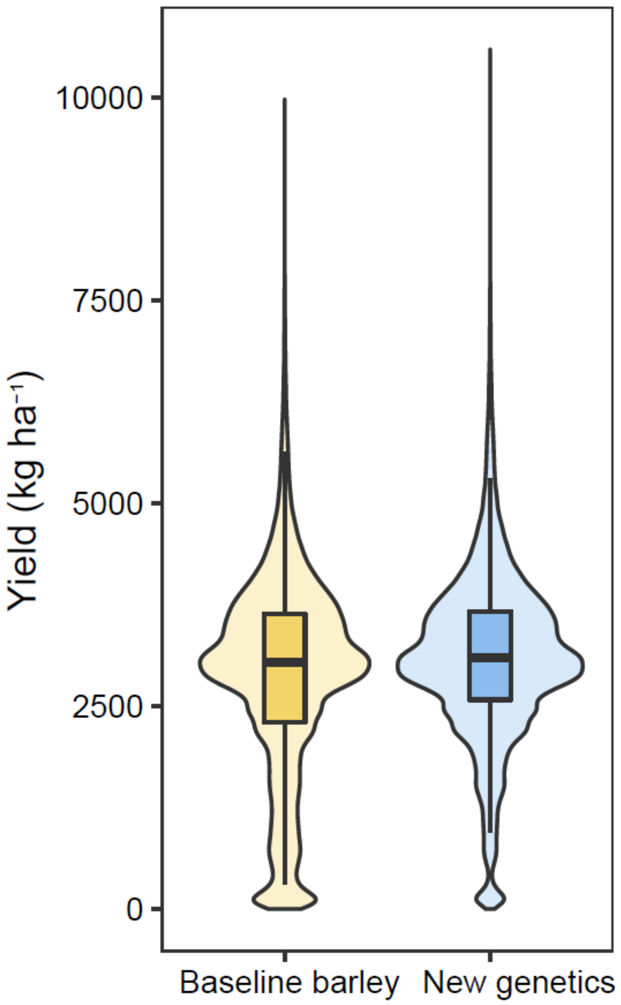

Supplementary Fig. 5| Simulated yield of barley genotypes with and without waterlogging tolerance genetics. Yield distributions were simulated under historical climates (1985-2016) across sites and sowing dates. Violin plots are plotted using the average of simulated values (1985-2016) at each site. The bottom, centre and top lines of the box represent the 25th, median and 75th percentiles.

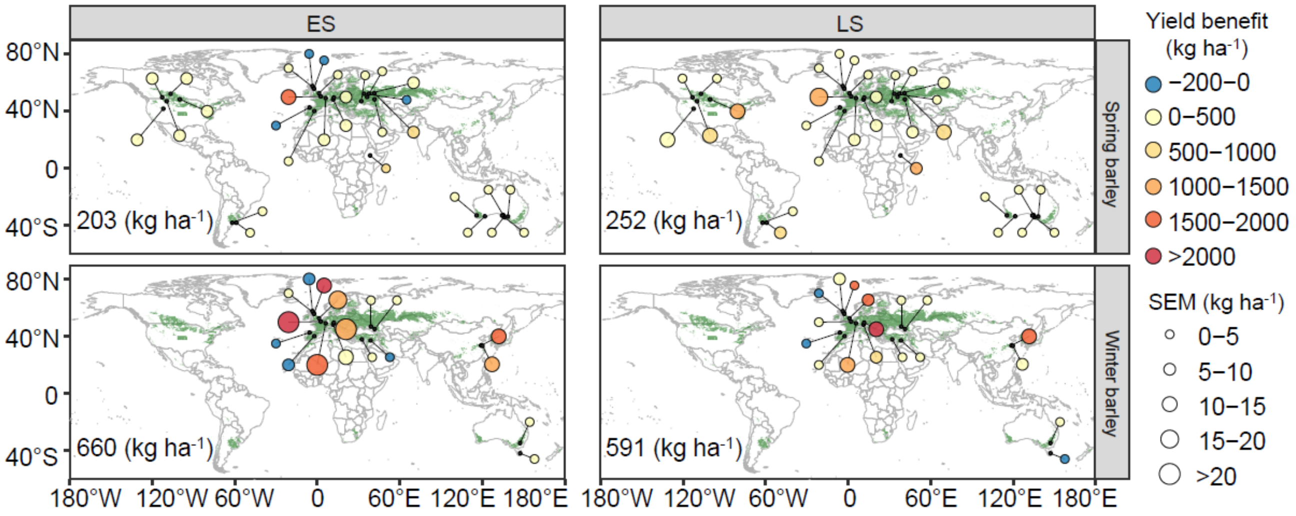

Supplementary Fig. 6| Mean and SEM of yield benefit associated with waterlogging tolerant barley genotypes compared with genotypes without waterlogging tolerance genes for the 2080 (2070-2099). Points are averaged across years and 27 GCMs in which growing season rainfall is higher than the 90th percentile; numerical values shown in each panel represent mean yield benefit across sites, years and GCMs.

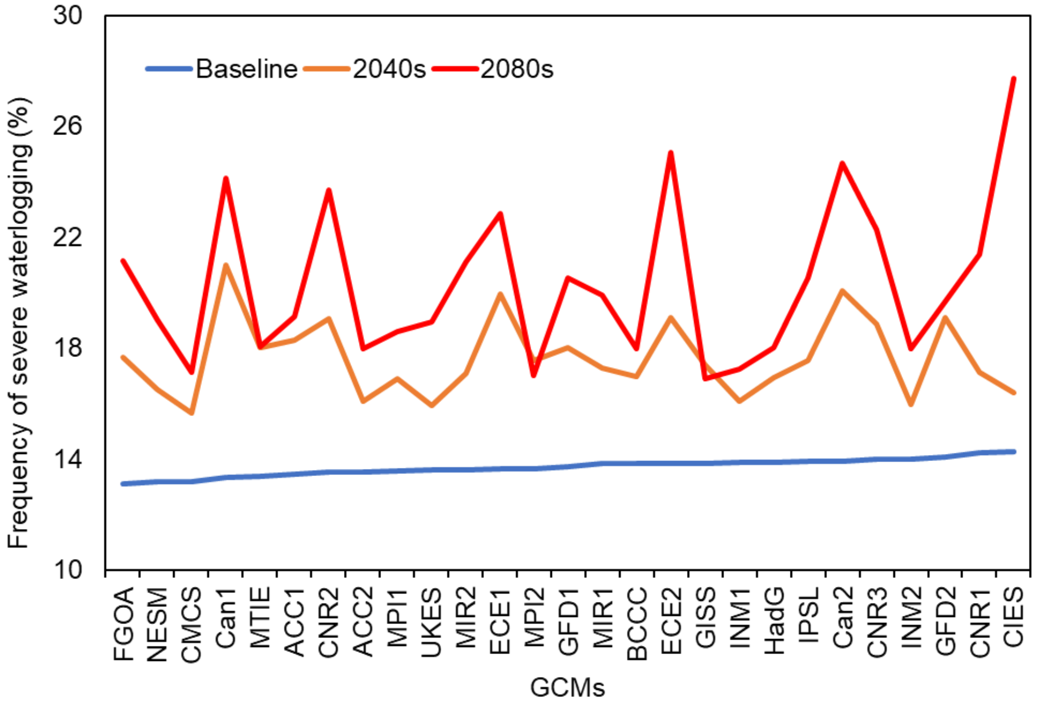

Supplementary Fig. 7| Projected risk of extreme waterlogging for each GCM for SSP585 for the baseline (1985-2016), 2040 (2030-2059) and 2080 (2070-2099). Risk of extreme waterlogging was computed as the sum of SW2 and SW3 for spring barley and WW2 and WW3 for winter barley (see Supplementary Fig. 3).

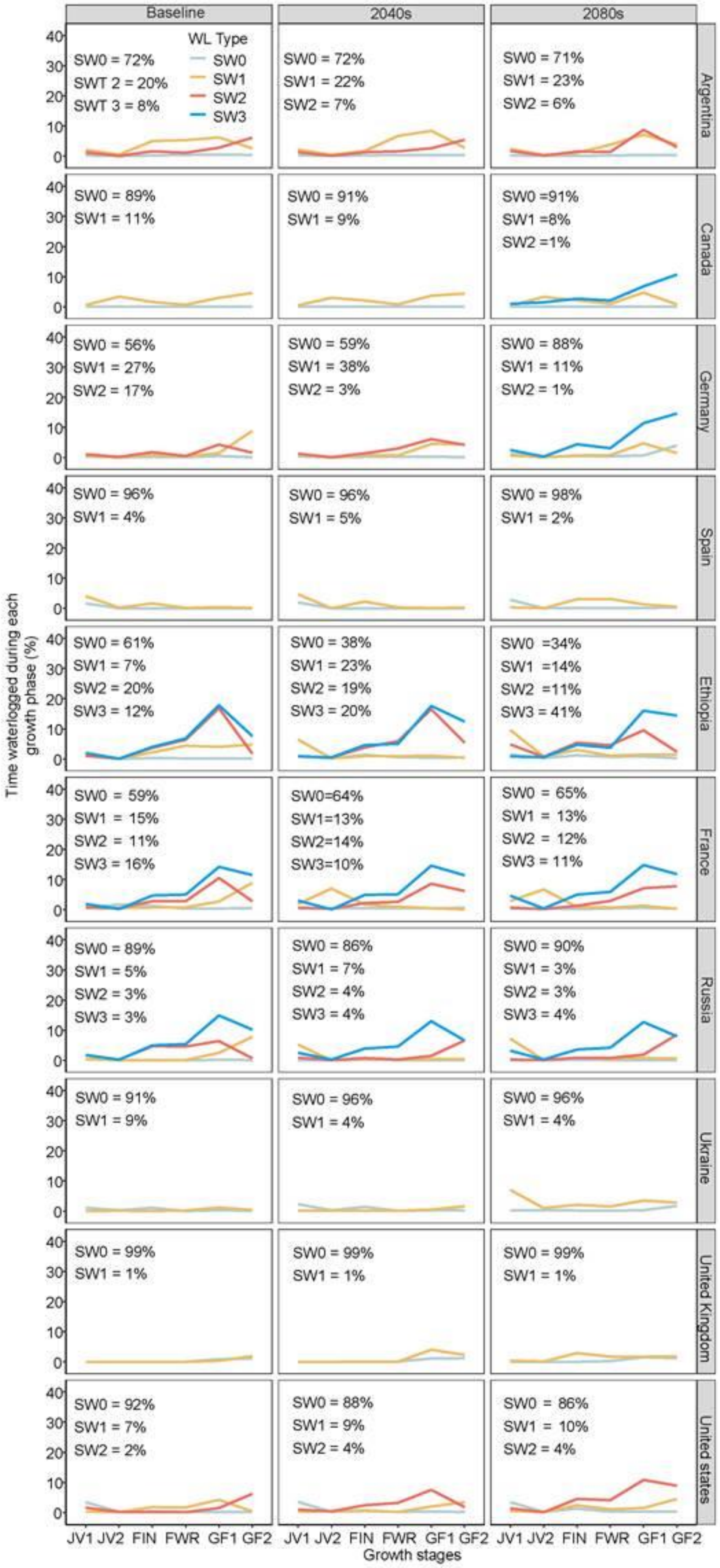

Baseline
2040s
2080s
WL Type
SW0
SW1
SW2
SW3
Argentina
SW0 = 72%
SWT 2 = 20%
SWT 3 = 8%
SW0 = 72%
SW1 = 22%
SW2 = 7%
SW0 = 71%
SW1 = 23%
SW2 = 6%
Canada
SW0 = 89%
SW1 = 11%
SW0 = 91%
SW1 = 9%
SW0 =91%
SW1 =8%
SW2 =1%
Germany
SW0 = 56%
SW1 = 27%
SW2 = 17%
SW0 = 59%
SW1 = 38%
SW2 = 3%
SW0 = 88%
SW1 = 11%
SW2 = 1%
Spain
SW0 = 96%
SW1 = 4%
SW0 = 96%
SW1 = 5%
SW0 = 98%
SW1 = 2%
Ethiopia
SW0 = 61%
SW1 = 7%
SW2 = 20%
SW3 = 12%
SW0 = 38%
SW1 = 23%
SW2 = 19%
SW3 = 20%
SW0 =34%
SW1 =14%
SW2 =11%
SW3 = 41%
France
SW0 = 59%
SW1 = 15%
SW2 = 11%
SW3 = 16%
SW0=64%
SW1=13%
SW2=14%
SW3=10%
SW0 = 65%
SW1 = 13%
SW2 = 12%
SW3 = 11%
Russia
SW0 = 89%
SW1 = 5%
SW2 = 3%
SW3 = 3%
SW0 = 86%
SW1 = 7%
SW2 = 4%
SW3 = 4%
SW0 = 90%
SW1 = 3%
SW2 = 3%
SW3 = 4%
Ukraine
SW0 = 91%
SW1 = 9%
SW0 = 96%
SW1 = 4%
SW0 = 96%
SW1 = 4%
United Kingdom
SW0 = 99%
SW1 = 1%
SW0 = 99%
SW1 = 1%
SW0 = 99%
SW1 = 1%
United states
SW0 = 92%
SW1 = 7%
SW2 = 2%
SW0 = 88%
SW1 = 9%
SW2 = 4%
SW0 = 86%
SW1 = 10%
SW2 = 4%
Time waterlogged during each growth phase (%)
0 10 20 30 40
JV1 JV2 FIN FWR GF1 GF2
Growth stages

Supplementary Fig. 8| Waterlogging stress patterns and frequencies for the baseline (1985-2016), 2040 (2030-2059) and 2080 (2070-2090) periods across countries, genotypes and sowing dates. Waterlogging stress patterns for spring barley include (SW0, low waterlogging; SW1: low moderate-late waterlogging; SW2; late-onset moderate waterlogging; SW3: late-onset severe waterlogging) and winter barley (WW0: low waterlogging; WW1: low early-onset waterlogging relieved later; WW2: moderate early-onset waterlogging; WW3: severe early-onset waterlogging). Stress patterns were identified by clustering seasonal time-courses of waterlogging stress across years, sowing dates, genotypes and countries. Growth stages include the early juvenile phase (JV1, 10<=APSIM growth stage<21); the late juvenile phase (JV2, 21<=APSIM growth stage<32); floral initiation to heading (FIN, 32<=APSIM growth stage<65); flowering to grain filling (FIN, 65<=APSIM growth stage<71; early grain filling (GF1, 71<=APSIM growth stage<80) and late grain filling (GF2, 80<=APSIM growth stage<87).

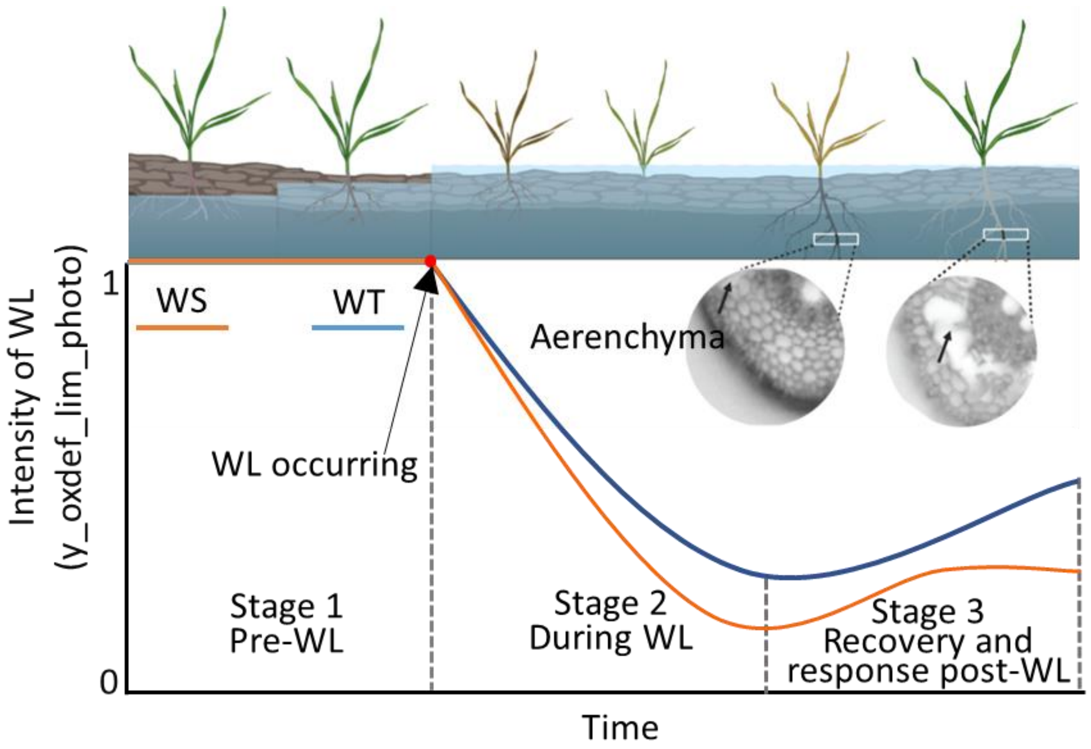

Supplementary Fig. 9| Conceptual design of crop response, adaptation and recovery from waterlogging for alternative genotypes (WS: waterlogging susceptible; WT: waterlogging tolerant).

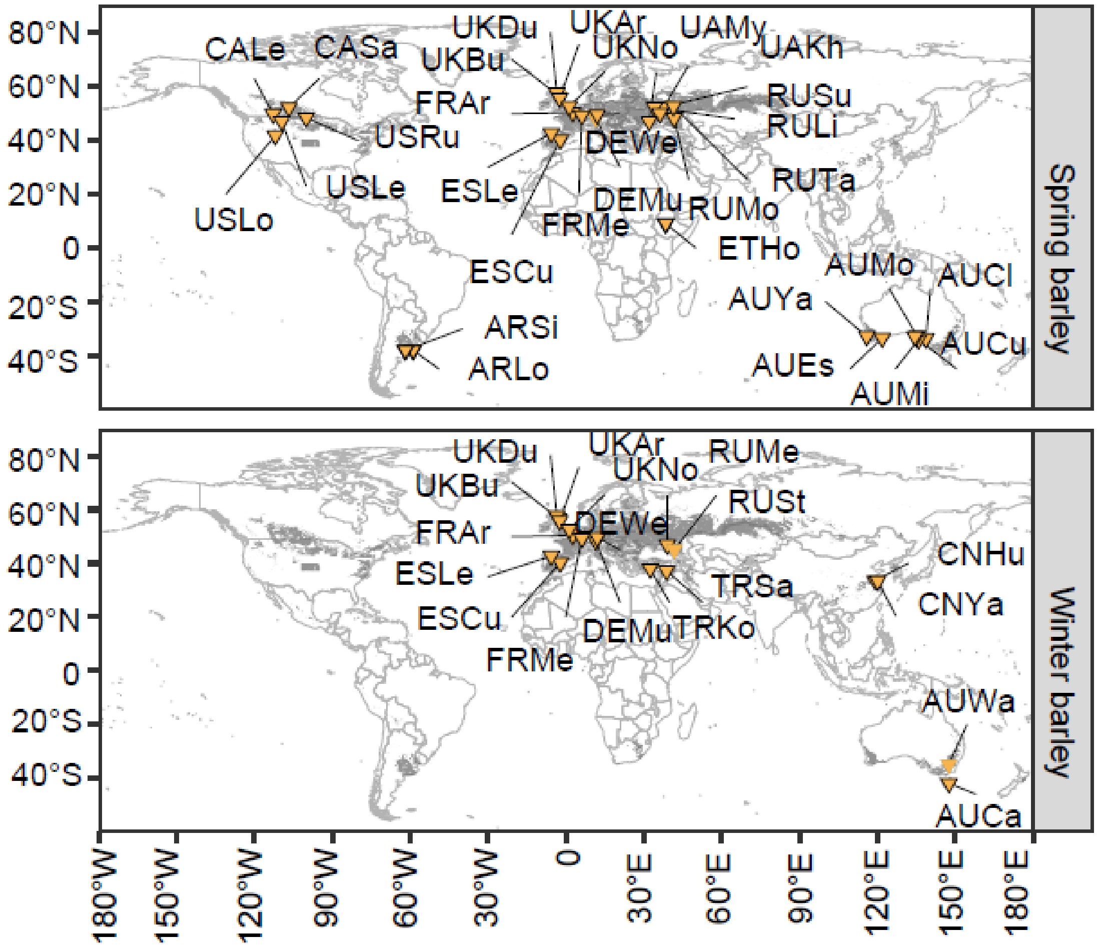

Supplementary Fig. 10| Study sites representing global barley production regions[8,9]. Point labels are abbreviations depicting country name concatenated with site name e.g. ARLo = Loberia, Argentina; ARSi = Sierra de La Ventana, Argentina; see Supplementary Table 1.

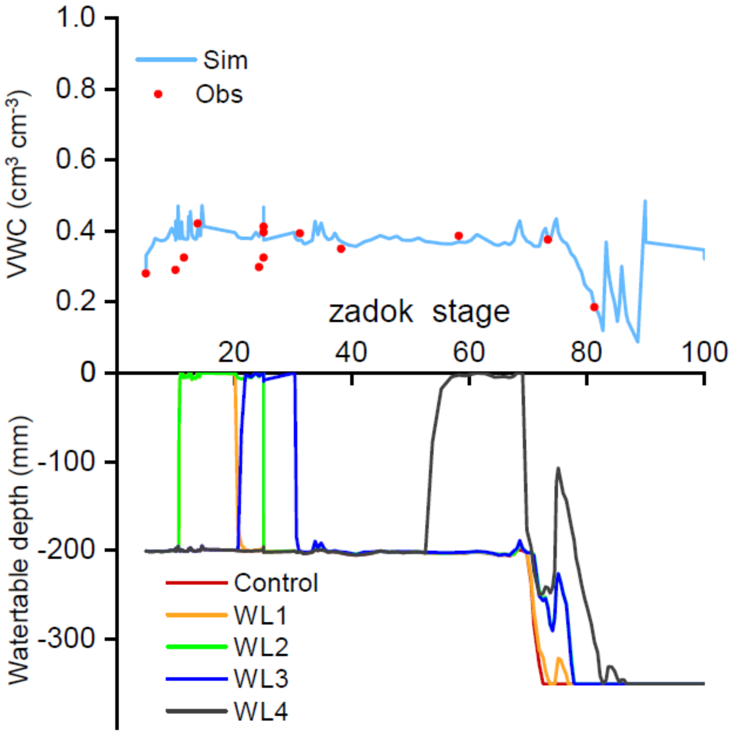

Supplementary Fig. 11| Simulated and observed volumetric soil water content (VWC) and water table dynamics with SWIM3 in APSIM. Experiments were conducted at Mt Pleasant Laboratories, Launceston, Tasmania, Australia[2]. **a,** diagram indicating the start and end date of each waterlogging treatment. WL1: waterlogging exposed at ZS12.5 for 1 month; WL2: waterlogging exposed at ZS12.5 for 2 months; WL3: waterlogging exposed at ZS15 for 2 months; WL4: waterlogging exposed at ZS59 for 15 days. WL4 treatment was not conducted on Franklin and Westminster. **b,** simulated and observed VWC and water table depth during the growing season.

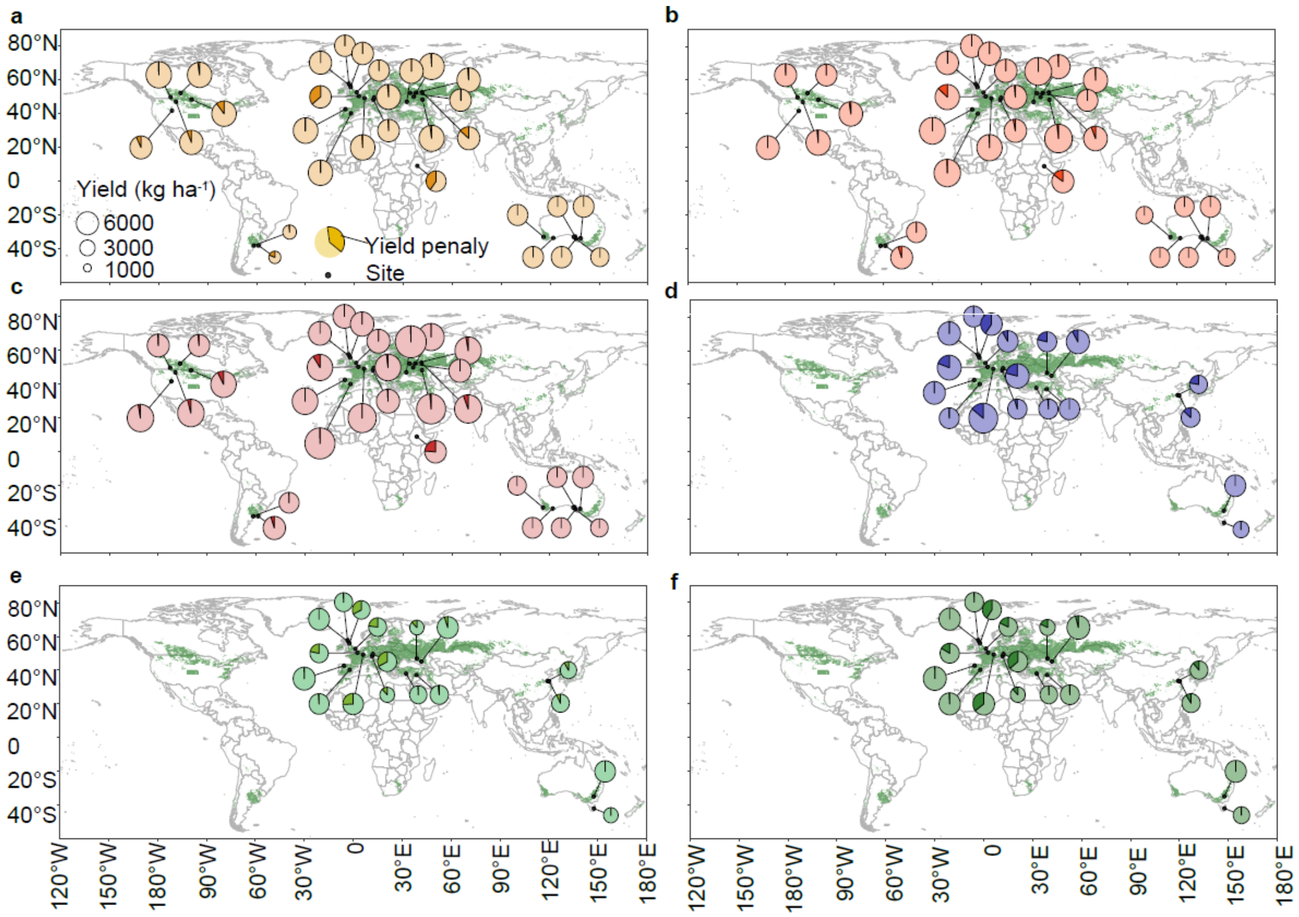

Supplementary Fig. 12 | Simulated yields for 2040 (2030-2059) and 2080 (2070-2099) under early (ES) or late sowing (LS). **a-f,** simulated yields (expressed as circles with two portions: yield penalty caused by waterlogging stress highlighted with darker shade) for spring barley under early sowing in 2040 (**a**), yields from spring barley under late sowing in 2040 (**b**) and 2080 (**c**), yields for winter barley under early sowing in 2040 (**d**), and simulated yields for winter barley under late sowing in 2040 (**e**) and 2080 (**f**). Yields were simulated with APSIM using downscaled data from 27 GCMs. Green regions on each map depict predominant barley cropping regions.

Supplementary References

1. Liu K, Harrison MT, Archontoulis SV, Huth N, Yang R, Liu DL*, et al.* Climate change shifts forward flowering and reduces crop waterlogging stress. *Environmental Research Letters* 2021, **16**(9)**:** 094017.

2. Liu K, Harrison MT, Ibrahim A, Manik SN, Johnson P, Tian X*, et al.* Genetic factors increasing barley grain yields under soil waterlogging. *Food and Energy Security* 2020, **9**(4)**:** e238.

3. de San Celedonio RP, Abeledo LG, Miralles DJ. Identifying the critical period for waterlogging on yield and its components in wheat and barley. *Plant and Soil* 2014, **378**(1)**:** 265-277.

4. Borrego-Benjumea A, Carter A, Zhu M, Tucker JR, Zhou M, Badea A. Genome-Wide Association Study of Waterlogging Tolerance in Barley (Hordeum vulgare L.) Under Controlled Field Conditions. 2021, **12**.

5. Byrne T, Grant J, Kock-Appelgren P, Förster L, Michel T, Miricescu A*, et al.* Improving phenotyping in winter barley cultivars towards waterlogging tolerance by combining field trials under natural conditions with controlled growth condition experiments. *European Journal of Agronomy* 2022, **133:** 126432.

6. Xue D, Zhou M, Zhang X, Chen S, Wei K, Zeng F*, et al.* Identification of QTLs for yield and yield components of barley under different growth conditions. *J Zhejiang Univ Sci B* 2010, **11**(3)**:** 169-176.

7. Xiao Y, Wei K, Chen J, Zhou M, Zhang G. Genotypic Difference in Growth Inhibition and Yield Loss of Barley under Waterlogging Stress. *Journal of Zhejiang University (Agric & Life Sci)* 2007, **33**(5)**:** 525-532.

8. International Food Policy Research Institute. Global spatially-disaggregated crop production statistics data for 2010 version 1.0. 2019.

9. Stackhouse PWJ, Westberg D, Hoell JM, Chandler WS, T Z. Prediction of world-wide energy resource (POWER)—Agroclimatology methodology—(1.0° latitude by 1.0° longitude spatial resolution). Hampton, NASA Langely Research Center. 2015.
