# Supplementary material for: Silver lining to a climate crisis in multiple prospects for alleviating crop waterlogging under future climates
Source: Nat Commun. 2023 Feb 10;14:765. doi: 10.1038/s41467-023-36129-4 (PMC9918449; doi:10.1038/s41467-023-36129-4)
Supplement: Supplementary file 2 — Reporting Summary [file 41467_2023_36129_MOESM2_ESM.pdf]

nature portfolio

Corresponding author(s): Matthew Tom Harrison

Last updated by author(s): Dec 16, 2022

Reporting Summary

Nature Portfolio wishes to improve the reproducibility of the work that we publish. This form provides structure for consistency and transparency in reporting. For further information on Nature Portfolio policies, see our Editorial Policies and the Editorial Policy Checklist.

Statistics

For all statistical analyses, confirm that the following items are present in the figure legend, table legend, main text, or Methods section.

n/a	Confirmed	
☐	☒	The exact sample size (*n*) for each experimental group/condition, given as a discrete number and unit of measurement
☒	☐	A statement on whether measurements were taken from distinct samples or whether the same sample was measured repeatedly
☐	☒	The statistical test(s) used AND whether they are one- or two-sided<br>*Only common tests should be described solely by name; describe more complex techniques in the Methods section.*
☒	☐	A description of all covariates tested
☒	☐	A description of any assumptions or corrections, such as tests of normality and adjustment for multiple comparisons
☐	☒	A full description of the statistical parameters including central tendency (e.g. means) or other basic estimates (e.g. regression coefficient) AND variation (e.g. standard deviation) or associated estimates of uncertainty (e.g. confidence intervals)
☐	☒	For null hypothesis testing, the test statistic (e.g. *F*, *t*, *r*) with confidence intervals, effect sizes, degrees of freedom and *P* value noted<br>*Give P values as exact values whenever suitable.*
☒	☐	For Bayesian analysis, information on the choice of priors and Markov chain Monte Carlo settings
☒	☐	For hierarchical and complex designs, identification of the appropriate level for tests and full reporting of outcomes
☒	☐	Estimates of effect sizes (e.g. Cohen's *d*, Pearson's *r*), indicating how they were calculated

Our web collection on statistics for biologists contains articles on many of the points above.

Software and code

Policy information about availability of computer code

Data collection	We used APSIM version 7.9 to conduct all simulations. All data and code used to conduct this study are available here: https://github.com/KeLiu7/Waterlogging-Barley
Data analysis	Computer codes for characterising common waterlogging stress patterns are provided on Github (https://github.com/KeLiu7/Waterlogging-Barley).<br><br>Clustering was applied using the R statistical package ‘stats’ (version 4.2.1, R Development Core Team, 2013), with clusters being defined such that total within-cluster variation was minimised (partitioning n observations into k clusters (the value of K is assigned as four) where each observation belongs to the cluster with the nearest mean, i.e. the cluster centroid).<br><br>The map was modified using R package ggplot2 ’maps (version 3.4.0)’ with the Natural Earth dataset in a publica domain (https://www.naturalearthdata.com).

For manuscripts utilizing custom algorithms or software that are central to the research but not yet described in published literature, software must be made available to editors and reviewers. We strongly encourage code deposition in a community repository (e.g. GitHub). See the Nature Portfolio guidelines for submitting code & software for further information.

Data

Policy information about availability of data

All manuscripts must include a data availability statement. This statement should provide the following information, where applicable:

- Accession codes, unique identifiers, or web links for publicly available datasets
- A description of any restrictions on data availability
- For clinical datasets or third party data, please ensure that the statement adheres to our policy

Simulated yield data are available in Supplementary Source data and genotypic parameters used in APSIM are available in Supplementary Table 3. Soil data and downscaled climate datasets are available online (https://github.com/KeLiu7/Waterlogging-Barley).

Human research participants

Policy information about studies involving human research participants and Sex and Gender in Research.

Reporting on sex and gender	N/A
Population characteristics	N/A
Recruitment	N/A
Ethics oversight	N/A

Note that full information on the approval of the study protocol must also be provided in the manuscript.

Field-specific reporting

Please select the one below that is the best fit for your research. If you are not sure, read the appropriate sections before making your selection.

☐ Life sciences ☐ Behavioural & social sciences ☒ Ecological, evolutionary & environmental sciences

For a reference copy of the document with all sections, see nature.com/documents/nr-reporting-summary-flat.pdf

Ecological, evolutionary & environmental sciences study design

All studies must disclose on these points even when the disclosure is negative.

Study description	This study combined genetic, physiological and biophysical knowledge to evaluate the impact of waterlogging stress on barley production on a global scale. We also assess genotypic and management prospects for alleviating crop waterlogging under future climates. First, we develop a method that provides new insight into common stress patterns across sites, genotypes, climate projections and management. We then applied the improved model and clustering paradigm for major barley production zones across the world with the specific objectives of (1) quantifying effects of climate change on waterlogging, (2) characterising common waterlogging stress patterns and frequencies across environments, (3) determining the extent to which common stress patterns change under future climates, (4) quantifying the extent to which waterlogging tolerance genotypes, genotypic phenology and sowing time mitigate effects of waterlogging under future climates. Our method for clustering stress trajectories into common groups could be applied generically to any crop improvement programme or study. Measured data used for model development and validation was obtained from previous experiments conducted in five countries under field and/or controlled conditions (Exp1-Exp5).
Research sample	To validate our new algorithms across a broad array of global cropping environments, we collected measured data from five previous two-year barley waterlogging experiments (details are described in the Data collection below). These represent contemporary barley genotypes with varying waterlogging tolerance (n=36). The selection criteria is that experiments were conducted with two years at least, and recorded waterlogging timing and duration, phenology, yield variance and soil water dynamic across genotypes and treatments.
Sampling strategy	N/A
Data collection	MXZ provided the barley germplasm and KL collected datasets for model development and validation in this study. Part of the data used (e.g. Exp2-5) was from previous studies (for details, see "ref52 (https://doi.org/10.1007/s11104-014-2028-6)" for Exp2, "ref53 (https://doi.org/10.3389/fpls.2021.711654)" for Exp3, "ref54 (https://doi.org/10.1016/j.eja.2021.126432)" for Exp4 and "ref55 (https://doi.org/10.1631/jzus.B0900332)" for Exp5. Measured data from five previous two-year experiments (Exp1, Exp2, Exp3, Exp4, Exp5) conducted in five countries (Australia, Argentina, Canada, China and Ireland) were used for watelogging calibration and validation. Exp1 was conducted under controlled conditions (Mt Pleasant Laboratories, Launceston, Tasmania, Australia) with four waterlogging treatments using six contemporary Australian barley genotypes differing in their waterlogging tolerance from 2019 to 2020 (see refs 34, 35). In Exp2, barley yields were measured under five waterlogging treatments in the greenhouse and field conditions at the School of Agronomy, University of Buenos Aires, Argentina during 2010 (see ref.52). In Exp3, barley genotypes were evaluated for waterlogging tolerance in controlled field conditions at Brandon Research and Development Centre, Brandon,

	Manitoba, Canada from 2016 to 2017. Waterlogging treatments were initiated at the tillering stage by adding the water to heights of 0.5–1 cm above the soil surface (see ref.53). In Exp4, barley yields were measured in field conditions carried out at Oak Park, Carlow, Ireland from 2017 to 2018. Waterlogging treatments were initiated at the tillering stage using a boom irrigator (see ref.54). In Exp5, field experiments were conducted in 2003-2004 and 2005-2006 at Zhejiang University, Hangzhou, China. Waterlogging treatments were imposed at tillering (see ref.55, 56). All experiments were carefully managed to provide adequate nutrition and control of biotic pressures.<br><br>KL downloaded the historical daily weather datasets (1985-2016) (e.g. rainfall, maximum and minimum temperature, solar radiation) from NASA Power (https://power.larc.nasa.gov/data-access-viewer/). Future climate datasets were downscaled with 27 GCMS by DLL. Soil parameters and management settings were prescribed constant values for all climate horizons. Soil parameters (soil texture, bulk density, pH, and organic carbon content etc) were obtained from the International Soil Reference and Information Centre (https://www.isric.org/explore/soilgrids, ref64).
Timing and spatial scale	Here, we simulated crop growth and development using the most plausible greenhouse gas emissions scenario (ie. SSP585) for climate periods of 2030-2059 and 2070-2099, relative to the historical baseline (1985-2016). Global simulations were conducted using 38 sites located in thirteen countries based on their national barley production and planting area (https://ipad.fas.usda.gov/rssiws/al/global_cropprod.aspx). These representative sites where barley is grown have sites with documented reports of waterlogging (ref 58 and ref59). In each country, simulated sites were prioritised based on dominant soil types in cropping zones from the Digital Soil Map of the World (https://www.fao.org/soils-portal/data-hub/soil-maps-and-databases/faounesco-soil-map-of-the-world/en/, ref 57).
Data exclusions	No data were excluded
Reproducibility	We believe that our study can be reproduced following the steps outlined in the methods and supplementary information.
Randomization	N/A
Blinding	N/A

Did the study involve field work? ☐ Yes ☒ No

Reporting for specific materials, systems and methods

We require information from authors about some types of materials, experimental systems and methods used in many studies. Here, indicate whether each material, system or method listed is relevant to your study. If you are not sure if a list item applies to your research, read the appropriate section before selecting a response.

Materials & experimental systems

n/a	Involved in the study
☒	☐ Antibodies
☒	☐ Eukaryotic cell lines
☒	☐ Palaeontology and archaeology
☒	☐ Animals and other organisms
☒	☐ Clinical data
☒	☐ Dual use research of concern

Methods

n/a	Involved in the study
☒	☐ ChIP-seq
☒	☐ Flow cytometry
☒	☐ MRI-based neuroimaging
